# Amelogenesis Imperfecta in Two Families with Defined *AMELX* Deletions in *ARHGAP6*

**DOI:** 10.1371/journal.pone.0052052

**Published:** 2012-12-14

**Authors:** Jan C.-C. Hu, Hui-Chen Chan, Stephen G. Simmer, Figen Seymen, Amelia S. Richardson, Yuanyuan Hu, Rachel N. Milkovich, Ninna M. R. P. Estrella, Mine Yildirim, Merve Bayram, Chiung-Fen Chen, James P. Simmer

**Affiliations:** 1 Department of Biologic and Materials Sciences, University of Michigan School of Dentistry, Ann Arbor, Michigan, United States of America; 2 Department of Pedodontics, Faculty of Dentistry, University of Istanbul, Istanbul, Turkey; 3 Department of Orthodontics and Pediatric Dentistry, University of Michigan School of Dentistry, Ann Arbor, Michigan, United States of America; Institut Jacques Monod, France

## Abstract

*Amelogenesis imperfecta* (AI) is a group of inherited conditions featuring isolated enamel malformations. About 5% of AI cases show an X-linked pattern of inheritance, which are caused by mutations in *AMELX*. In humans there are two, non-allelic amelogenin genes: *AMELX* (Xp22.3) and *AMELY* (Yp11.2). About 90% of amelogenin expression is from *AMELX*, which is nested within intron 1 of the gene encoding Rho GTPase activating protein 6 (*ARHGAP6*). We recruited two AI families and determined that their disease-causing mutations were partial deletions in *ARHGAP6* that completely deleted *AMELX*. Affected males in both families had a distinctive enamel phenotype resembling “snow-capped” teeth. The 96,240 bp deletion in family 1 was confined to intron 1 of *ARHGAP6* (g.302534_398773del96240), but removed alternative *ARHGAP6* promoters 1c and 1d. Analyses of developing teeth in mice showed that *ARHGAP6* is not expressed from these promoters in ameloblasts. The 52,654 bp deletion in family 2 (g.363924_416577del52654insA) removed *ARHGAP6* promoter 1d and exon 2, precluding normal expression of ARHGAP6. The male proband of family 2 had slightly thinner enamel with greater surface roughness, but exhibited the same pattern of enamel malformations characteristic of males in family 1, which themselves showed minor variations in their enamel phenotypes. We conclude that the enamel defects in both families were caused by amelogenin insufficiency, that deletion of *AMELX* results in males with a characteristic snow-capped enamel phenotype, and failed *ARHGAP6* expression did not appreciably alter the severity of enamel defects when *AMELX* was absent.

## Introduction

Non-syndromic amelogenesis imperfecta (AI) is a heterogeneous collection of inherited defects in dental enamel formation that includes a multiplicity of enamel phenotypes, patterns of inheritance, and causative genes [1], [2]. Non-syndromic forms of AI are divided into 14 subtypes [3]. To date, defects in 7 genes have been show to cause non-syndromic AI. Autosomal dominant forms can be caused by defects in enamelin (*ENAM*, 4q21) [4] and family with sequence similarity 83 member H (*FAM83H*, 8q24.3) [5]. Autosomal recessive AI can be caused by defects in enamelysin (*MMP20*, 11q22.3-q23) [6], kallikrein-related peptidase 4 (*KLK4*, 19q13.4) [7], WD repeat containing domain 72 (*WDR72*, 15q21.3) [8] and chromosome 4 open reading frame 26 (C4orf26, 4q21.1) [9]. X-linked AI is caused by amelogenin defects (*AMELX*, Xp22.31-p22.1) [10]. Together, defects in these genes account for about half of all AI cases [11], [12]. X-linked AI accounts for about 5% of all AI cases [13]. There are two amelogenin genes (*AMELX* and *AMELY*), but the gene on the Y-chromosome (Yp11.2) is expressed at low levels [14]. *AMELX* and *AMELY* do not undergo homologous recombination, have diverged [15], and because of this divergence, are able to be used in forensics for sex determination [16]. The wide scale application of PCR tests for sex determination provides data on the frequency of *AMELY* deletions. DNA repeat sequences on the Y chromosome apparently create a structural instability that leads to recurrent 3–4 Mb deletions inclusive of *AMELY*, *PRKY* and *TBL1Y*, and sometimes *PCDH11Y,* with no apparent negative selection [17]. The frequency of *AMELY* deletions varies by population and is generally low, perhaps about 0.6% [18]. It is ∼0.02% in Australia [19] and ∼0.04% in China [20], but higher among particular ethnic groups (∼3.6%) in Malaysia and India [21]. *AMELY* deletions have been reported for “normal populations”, with the extrapolation that the tooth enamel must therefore be normal in these people. To our knowledge no oral photos or radiographs have been published of person lacking *AMELY*, although two individuals with *AMELY* deletions were specifically reported to have normal teeth [22].

Amelogenin (*AMEL*) belongs to the secretory calcium-binding phosphoprotein (SCPP) family of genes [23]. With the exception of *AMEL*, all human SCPP genes are clustered on chromosome 4, which include 5 genes encoding acidic proteins and 16 genes encoding proteins enriched proline and glutamine [24]. *Amel* apparently arose in the proline-glutamine-rich gene cluster and later transposed into intron 1 of *Arhgap6* (Rho GTPase activating protein 6) in the opposite orientation, resulting in a nested gene structure. This may have occurred in fish, as *Amel* is already nested in *Arhgap6* in African clawed toads, suggesting that such was the localization of *Amel* in early tetrapods [25]. In eutherian mammals the pair of homologous autosomes that carried these nested genes (*Amel* and *Arhgap6*) translocated to the sex chromosomes [25].

ARHGAP6 is a GTPase-activating protein of the Rho-GAP family. Mice deleted for a 1.1-Mb genomic region spanning from the first exon of *Arhgap6* (inclusive of *Amelx*) to the 5′end of *Mid1* did not cause any detectable phenotypic or behavioral abnormalities beyond dental enamel defects. The enamel malformations were largely the same as those observed in the *Amelx* null mice (thin enamel, ∼20 µm versus ∼115 µm, comprised of parallel-oriented crystallites) with the possible exception of a “flattened layer” on the enamel surface [26]. Despite this potential variation of the enamel phenotype in the *Arhgap6*/*Amelx* double null mice relative to the *Amelx* single nulls, two other *Arhgap6* knockouts that retained *Amelx* showed no enamel phenotype. A knockout deleting only exons 6–8 of *Arhgap6* resulted in mutant male and female mice “indistinguishable from their wild-type littermates at birth and thereafter” [27]. “Deletion from exon 1a of *Arhgap6* to *Mid1* led to enamel abnormalities but deletion from exon 6 of *Arhgap6* to *Mid1* did not” [26]. These findings suggest that *Arhgap6* is not essential for normal enamel formation, but might modify the enamel malformation phenotype when *Amelx* is absent.

In humans, larger X-chromosome deletions inclusive of *AMELX* cause syndromes that include AI as a phenotype [28]. Microphthalmia with linear skin defects syndrome (MLS; OMIM 309801) is caused, at least in part, by inactivating mitochondrial holocytochrome synthetase (*HCCS*) adjacent to *ARHGAP6* and *AMELX* on Xp. To date, 16 *AMELX* mutations have been reported that cause non-syndromic amelogenesis imperfecta (Fig. S1). A partial *AMELX* deletion (g.2525_7247del4723) starting in intron 2 and ending in exon 7 retained only the coding region for the amelogenin signal peptide plus 2 amino acids [29], [30]. The enamel phenotype of this family “B” [10] or “pedigree 41” [13], [31] was described as X-linked recessive hypomineralization AI. Affected women had vertically ridged teeth, particularly in the anteriors, which were attributed to alternating bands of normal and hypoplastic enamel deposited by ameloblasts that had randomly inactivated either the normal or defective X-chromosome during development [32]. Affected males had a more severe enamel phenotype.

In this study we report the characterization of two kindreds with partial *ARHGAP6* deletions that remove all of *AMELX*. In family 1 the deletion is confined to intron 1 and is not predicted to alter *ARHGAP6* expression. In family 2 the deletion includes exon 2 of *ARHGAP6* and is predicted to eliminate normal *ARHGAP6* expression. These naturally occurring gene knockouts are analyzed to see what information can be gained concerning the human enamel phenotype in the absence of *AMELX* expression and the potential importance of *ARHGAP6* in the process of dental enamel formation.

## Materials and Methods

The human study protocol and subject consents were reviewed and approved by the Institutional Review Board at the University of Michigan and the Ethics Committee of Istanbul University, Turkey, where one of the families was recruited. Study participants signed appropriate written consents after an explanation of their contents and after their questions about the study were answered. Any minors age 8 or older signed a written assent form after their parent completed a written parental consent for participation of the minor. The animal protocol was reviewed and approved by the University Committee on Use and Care of Animals (UCUCA) at the University of Michigan.

### Characterization of Dental Phenotypes

The proband (III:4) of Family 1 was a 10-year-old dental patient at the University of Istanbul, Turkey. Oral photographs and radiographs were obtained for the proband and his 11-year-old cousin (III:1). The proband of Family 2 (III:7) was an 11-year-old dental patient at the Children's Clinic, University of Michigan School of Dentistry. His family was Caucasian, of Eastern European decent. Oral photographs and radiographs were obtained for the proband (III:7) and his mother (II:4). Tooth H of the proband was extracted for orthodontic considerations, photographed, and examined by scanning electron microscopy.

### DNA isolation, amplification, and sequencing

Peripheral whole blood (5 cc) or buccal swabs were obtained from the 8 members of Family 1 and 5 members of Family 2. Genomic DNA was isolated using the QIAamp DNA Blood Maxi Kit and protocol (Qiagen Inc, Valencia, CA) and its quality and quantity were determined by spectrophotometry at OD_260_ and OD_280_. Genomic DNA (50 ng) was amplified using the Platinum PCR Supermix (Invitrogen, Carlsbad, CA), and the amplification products were purified using the QIAquick PCR Purification Kit and protocol (Invitrogen, Carlsbad, CA). The concentration of purified amplicon was estimated by the intensity of its ethidium bromide-stained band on a 1% agarose gel. The DNA sequencing reactions used 3 ng/µL for each 1000 bp of amplification product size and 1.0 pMol/µL of oligonucleotide primer and were analyzed using an ABI Model 3700 DNA sequencer (Applied Biosystems, Foster City, CA) at the University of Michigan DNA sequencing core.

### Polymerase Chain Reaction and DNA Sequence Analyses

The seven *AMELX* exons along with adjoining intron sequences were amplified by polymerase chain reaction (PCR); however, amplification products were only observed for male and female controls–not from either proband (Fig. S2). We concluded *AMELX* was deleted in both probands. Because the families reported no notable medical histories and the clinical phenotype was limited to the enamel layer, we expected the deletions to be relatively small. PCR primer pairs were designed to survey upstream and downstream regions. Eight primer pairs sampled intron 1 of *ARHGAP6* from nucleotides 50,924 to 291,746 (NCBI genomic reference sequence NG_012494.1) (Fig. S3). PCR amplification products were observed from both probands in all reactions. *ARHGAP6* exons 2 through 7 were sampled on the other side of *AMELX*. Only exon 2 of *ARHGAP6* in family 2 failed to amplify (Fig. S3). Thus the deleted regions were confined to the 5′ region of *ARHGAP6*. Additional amplifications narrowed down the locations of the deletions in family 1 (Fig. S4) and family 2 (Figs. S5 and S6).

To sequence across the deletion in Family 1 genomic DNA was amplified with F: GCTAATTATTGGTGGAAAAG and Fa1-11R: GAACAGAGGCAGGCTGTGTC, producing a 2200 bp product. The amplification reactions included a 2 min denaturation at 94°C followed by 35 cycles at 94°C for 30 s, annealing at 57°C for 1 min, extension at 72°C for 6 min followed by a final extension for 20 min. The products were characterized by DNA sequencing priming with the primers used for amplification as well as Fa1-BPF: TTGCCAATCTGCTTTTTACAG, and Fa1-BPR: TATCCTCTTTGTGGGACAGC.

To sequence across the deletion in family 2, genomic DNA was amplified with Fa2-5F: TGAAAGCTAGAGGGGAAACC and Fa2-10R: TGCCAAGAGTAGCCATTTGA, producing a 1969 bp product. The amplifcation reactions included a 5 min denaturation at 94°C followed by 35 cycles at 94°C for 90 s, annealing at 58°C for 1 min, extension at 72°C for 3 min, followed by a final extension for 7 min.

### Scanning electron microscopy (SEM)

The primary tooth (H) from the proband of Family 2 was sputter coated with gold for 75 s and then imaged using a Field Emission Gun Scanning Electron Microscope (FEG-SEM; Amray 1910 Field Emission Scanning Electron Microscope) at the Microscopy and Image Analysis Laboratory at the University of Michigan.

### *Arhgap6* RT-PCR in mouse molars

To determine which of the four *Arhgap6* promoters (1A–1D) are used during tooth development, day 5 and day 11 developing first mandibular molars were collected from C57BL6 wild-type mice and processed according to the published protocol [33]. Secretory and maturation stage ameloblasts were obtained by laser capture microdissection (LCM) from the cusp slopes of ∼30×8 µM sections of day 5 and day 12 maxillary first molars, respectively. This was accomplished using a Leica AS LMD (Leica Microsystems, Wetzlar, DE). The cuttings dropped by gravity into PCR test tubes containing RNA extraction buffer. RNA was extracted using an Arcturus PicoPure RNA Isolation Kit (Life Technologies, Carlsbad CA, USA). Then 16.5 ng of RNA was converted into cDNA using Invitrogen's VILO cDNA Synthesis Kit (Life Technologies). RNA was extracted from spleen, lung, and enamel organ epithelium (EOE) of day 5 and day 12 mouse mandibular first molars for positive PCR controls. The PCR reactions used promoter-specific forward primers (Ex1aF: GCAAGCATCCTCAGTTCCTC; Ex1bF: GCAGTGAAGTAAGGGGACCA; Ex1cF: GTGACTCCTAGGGGACCACA; Ex1dF: AAGACAGCAAAGACACCGAGA) paired with an exon 4-specific reverse primer (Ex4R: GGGATAAGGGCATTCCAAAT). The PCR amplifications included a 5 min denaturation at 95°C followed by 35 cycles at 94°C for 30 s, annealing at 58°C for 30 s, extension at 72°C for 1 min. In the last cycle the extension was for 7 min. The products were analyzed on a 1% agarose gel stained with ethidium bromide.

## Results

**Figure 1:**
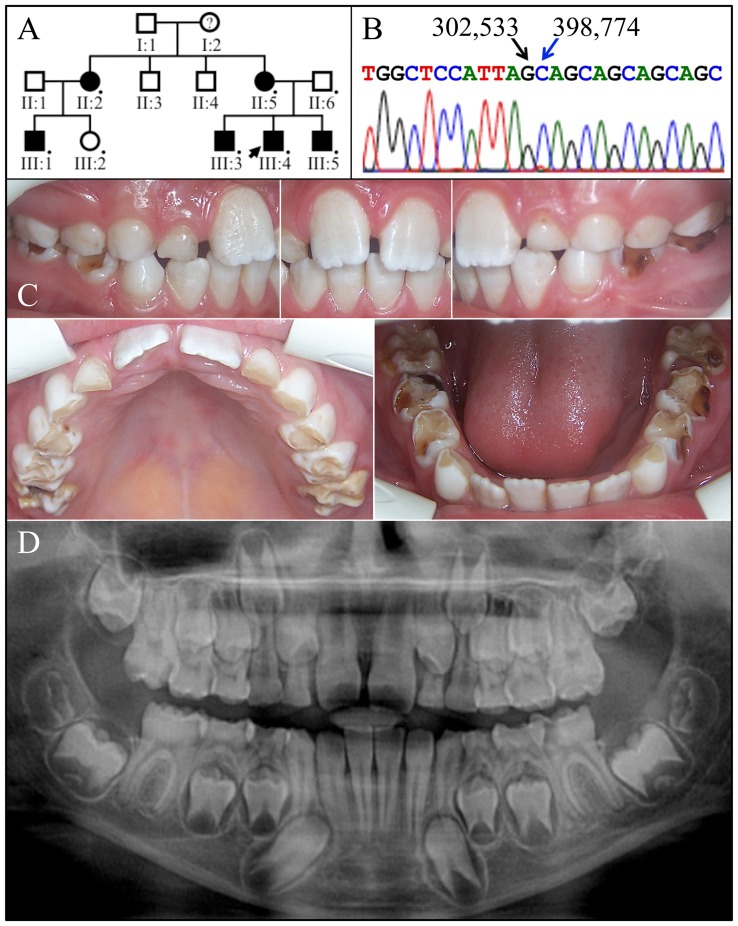
Family 1. ***A:*** Pedigree of family 1 consistent with an autosomal dominant or X-linked pattern of inheritance. DNA was obtained from 8 persons, each indicated by a black dot. The arrow identifies the proband. A question mark indicates that the enamel phenotype was unknown. ***B:*** The proband's (X*Y; III:4) DNA sequencing chromatogram spanning the deleted segment of *ARHGAP6* (g.302534_398773del96240) containing all of *AMELX*. ***C:*** Oral photographs of the proband, a 10 year old Caucasian male in the mixed dentition stage of dental development. Most of the occlusal enamel had abraded from the primary molars, revealing the thinness of retained enamel on the lateral and mesial/distal surfaces. ***D:*** Panoramic radiograph showing a thin layer of developing enamel on the unerupted bicuspids and second molars. The enamel is more radioopaque than dentin, but less so than normal enamel.

The proband (III:4) of family 1 was a 10-year-old Caucasian male from Turkey. His mixed dentition displayed thin enamel that was only slightly more radiopaque than dentin and in some locations secondarily affected by dental caries (Fig. 1). The occlusal surfaces of the primary molars, particularly the primary first molars, were completely worn through to dentin. The permanent mandibular central incisors had recently erupted and exhibited unusually thin enamel with a rough and pitted surface. The newly erupted first molars showed more enamel on the cusp tips than on the lateral and occlusal surfaces, giving them a “snow capped” appearance. The incisal edges of the maxillary permanent central incisors were also visibly whiter than the rest of the crown. The pedigree was consistent with an X-linked or autosomal dominant pattern of inheritance.

**Figure 2:**
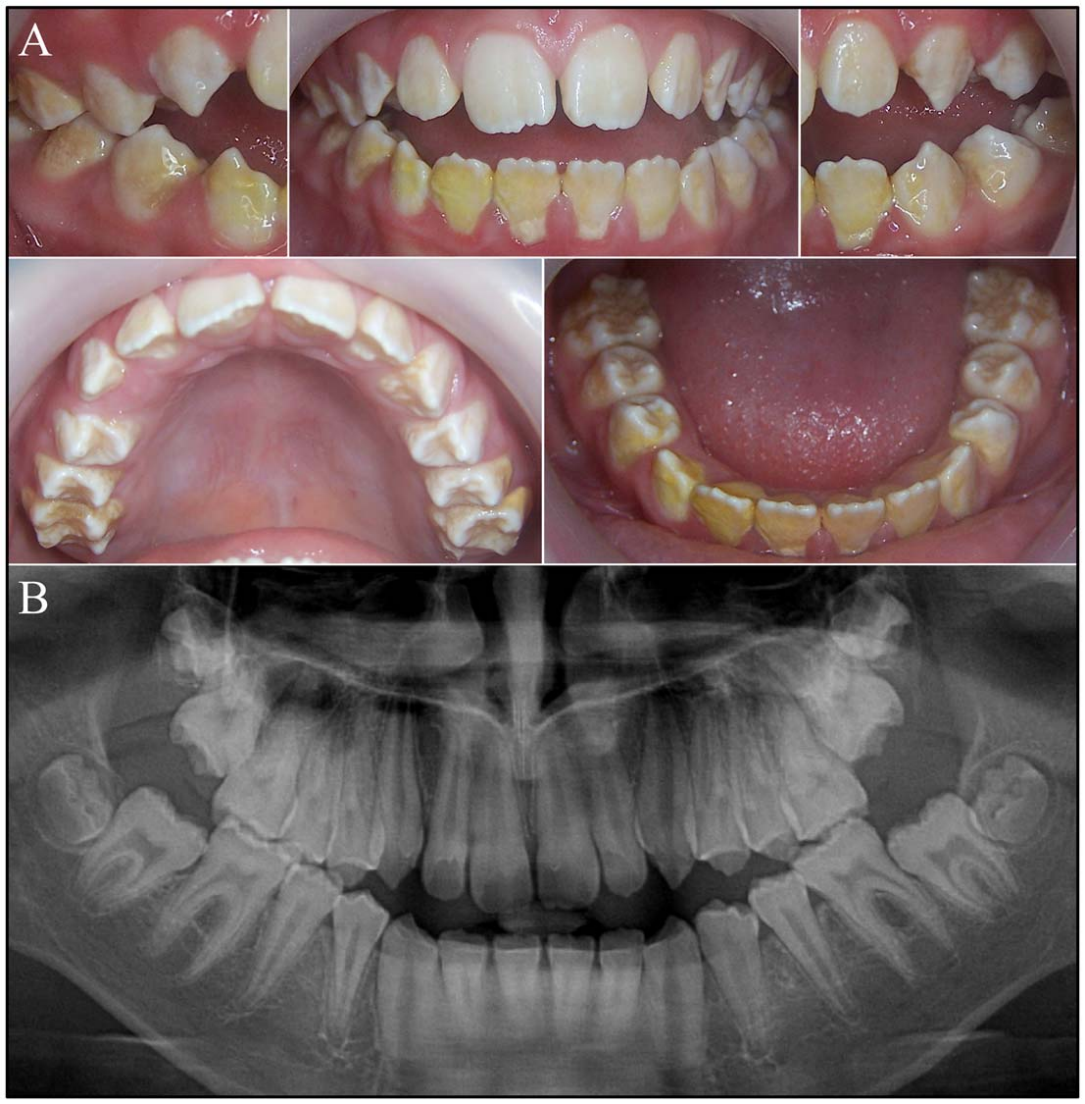
Family 1 cousin (X*Y; III:1, Fig. 1A) of the proband. ***A:*** Oral photographs of the proband's affected cousin with an anterior open bite at age 12. The enamel is whitest and thickest along the incisal edges and cusp tips. Enamel on the lateral surfaces is thin and rough and retains plaque. ***B:*** Panoramic radiograph reveals a thin layer of enamel on erupted and unerupted teeth that is more radioopaque than dentin, but less than normal enamel.

**Figure 3:**
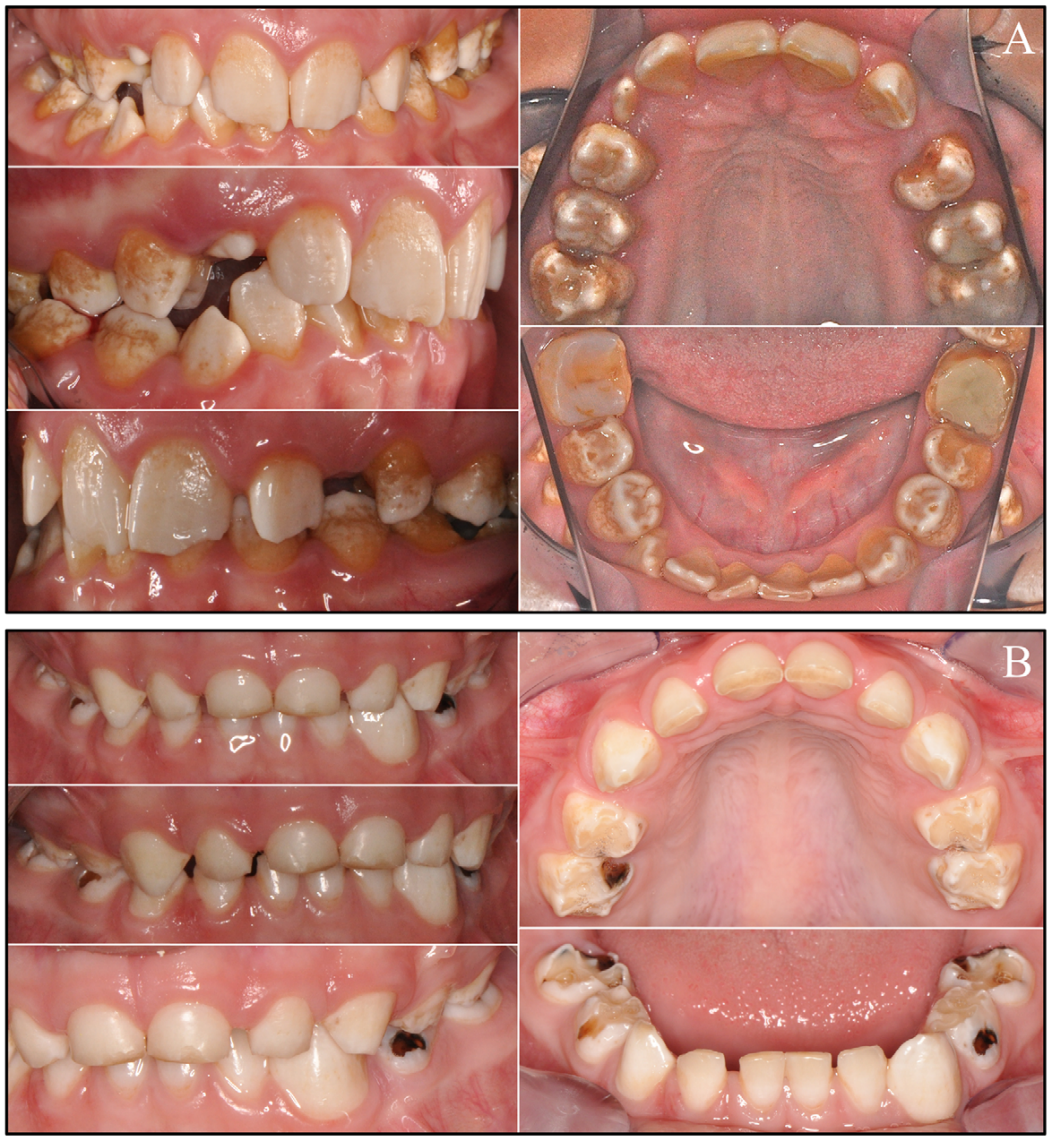
Oral photographs of the proband's older (12 year old) brother (III:3, Fig. 1A). All teeth are permanent (the secondary dentition). The enamel of the posterior teeth was thickest along the cusp tips and thinner on the lateral surfaces of the crowns, which were stained and showed the underlying dentin. Attrition is evident, particularly on the first molars and the enamel is chipped in many locations. ***B:*** Oral photographs of the proband's younger (4 year old) brother (III:5, Fig. 1A). All teeth are from the primary dentition. Attrition of the molar occlusal surfaces shows the thinness of the enamel. The incisal edges of the anterior teeth are worn and chipped. Some teeth are secondarily affected by dental caries.

The proband's affected male cousin (III:1) at age 12 exhibited enamel defects similar to those of the proband (Fig. 2), although he had an anterior open bite. His dentition showed small spacing between the teeth, and exhibited thin, rough, pitted enamel with extensive calculus build-up, particularly on the mandibular teeth. The enamel covering the cusp tips and incisal edges appeared to be whiter and less severely affected than the enamel on the lateral and occlusal surfaces. Radiographically the enamel layer was thin, but contrasted with dentin. The proband's 15 year old (III:3) and 4 year old (III:5) brothers were also affected (Fig. 3). The enamel of the primary dentition was thin, chipped easily, and showed pronounce attrition on working surfaces. The enamel of the secondary dentition was better formed on the incisal edges of the anterior teeth and the cusp tips and ridges of the posterior teeth, with progressively less enamel cervically. Based upon the darker color of the lateral surfaces of the posterior teeth of the 15 year old relative to those of his 10 and 12 year old relations, it appears that the enamel deteriorates relatively rapidly following eruption.

**Figure 4:**
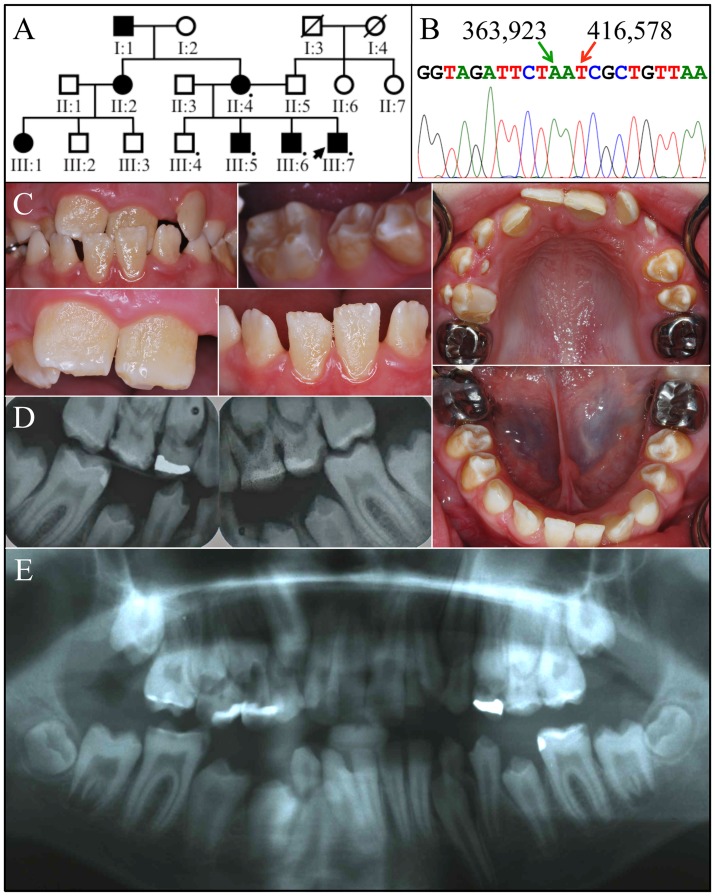
Family 2. ***A:*** Pedigree of family 2 consistent with an autosomal dominant or X-linked pattern of inheritance. DNA was obtained from 5 persons, each indicated by a black dot. The arrow identifies the proband. A diagonal line indicates the family member is deceased. ***B:*** DNA sequencing chromatogram spanning the deleted segment of *ARHGAP6* (g.363924_416577del52654insA) containing all of *AMELX* and exon 2 of *ARHGAP6*. ***C:*** Oral photographs of the proband, a 12 year old Caucasian male. The color of dentin showed through the thin enamel surfaces of the permanent central incisors, which had pitted, rough surfaces and whiter incisal edges. The enamel of the permanent first molars was thickest along the cusp tips and marginal ridges, and thinner on the occlusal and lateral surfaces. There were spaces between most teeth. ***D:*** Bitewing radiographs and ***E:*** a panoramic radiograph reveal a thin layer of enamel that is more radioopaque than dentin, but not as radiodense as normal enamel.

The proband of family 2 (III:7) was an 11-year-old Caucasian male of Eastern European ancestry with an anterior cross-bite. His late, mixed dentition displayed thin, rough enamel that barely contrasted with dentin on radiographs (Fig. 4). The incisal edges of the anterior teeth were whiter than the rest of the crowns and the posterior teeth had more enamel on the cusp tips than on the lateral surfaces. Thus, the distinctive enamel phenotype in the male subjects was a “snow-capped” appearance. Overall the character of the enamel defects was remarkably similar for the male members of the two families, although the proband of family 2 seemed to have greater surface roughness.

**Figure 5:**
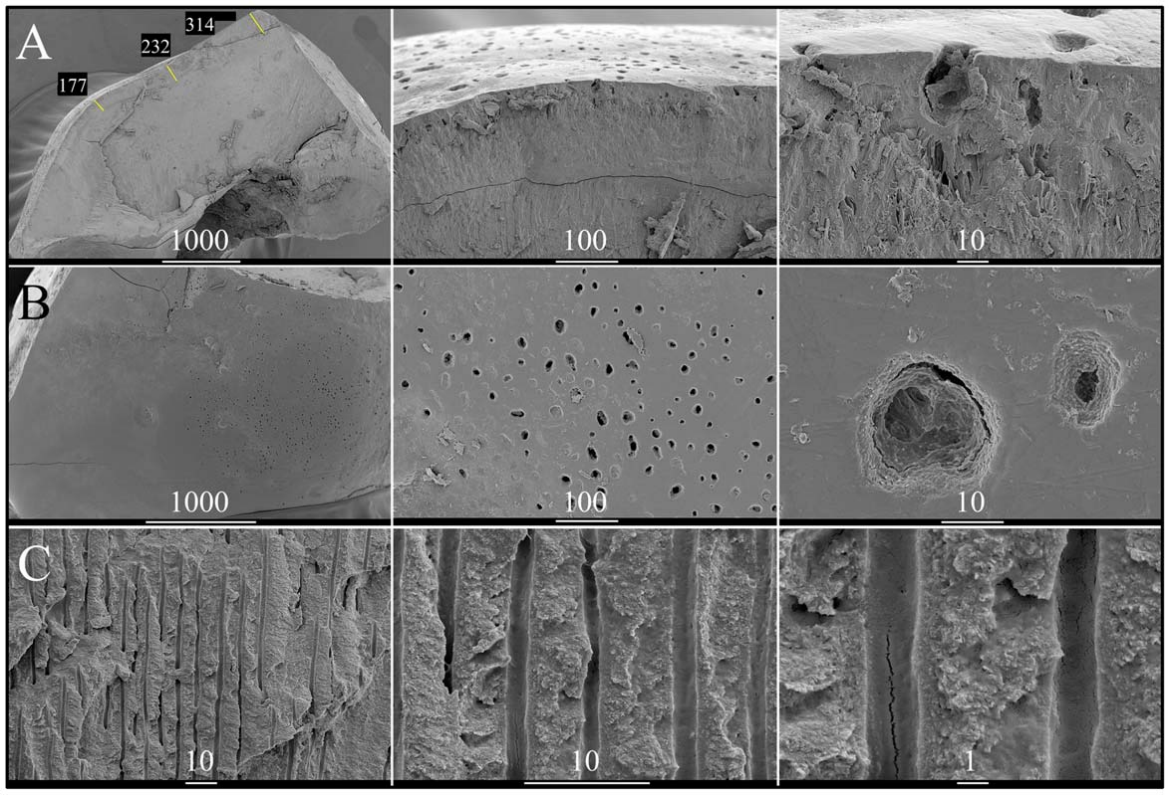
Scanning Electron Micrographs (SEMs) of the primary left maxillary cuspid (tooth H) from the proband of Family 2. ***A Left:*** Manually fractured surface of the cuspid showing the measured thickness of enamel at the cingulum and two positions moving up the cusp slope. ***A & B:*** The enamel surface appears to be smooth with extensive micro-pitting. ***C:*** Dentin with parallel dentinal tubules appears normal. Scale bar units are in µm.

A primary cuspid (Fig. S7; tooth H) extracted from the proband for orthodontic considerations was examined by scanning electron microscopy (Fig. 5). The enamel layer was readily distinguished from dentin and measured from 177 µm thick at the cingulum to 314 µm where the enamel still remained on the cusp slope in the direction of the cusp tip. This is well below the average thickness (1140 µm) of enamel on primary teeth [34]. The scanned surface of the enamel appeared smooth, but marked by craters roughly 10 µm to 50 µm in diameter that were not noticable without magnification. The dentin appears to be completely normal.

**Figure 6:**
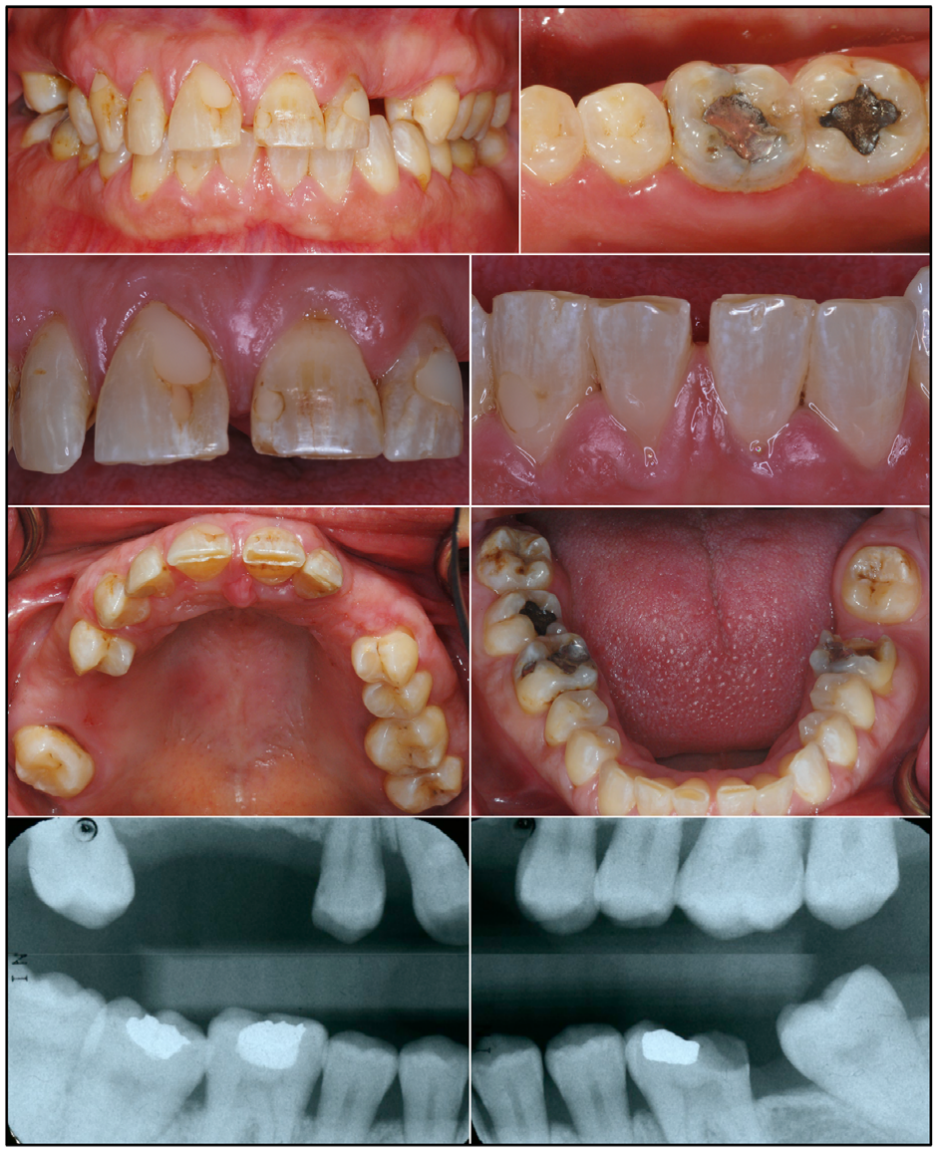
Mother (X*X; II:2, Fig. 4A) of family 2 proband. Oral photographs depicting multiple vertical cracks in incisor enamel and bitewing radiographs showing thin enamel that contrasts with dentin but is not as radioopaque as normal enamel.

The mother (II:4) of the proband was less severely affected than any of the male subjects (Fig. 6). Her enamel was thin and contrasted well with dentin on radiographs, but was not as radioopaque as normal. White flecks, similar to those associated with mild fluorisis, were observed in the anterior teeth, and were sometimes arranged in vertical columns.

**Figure 7:**
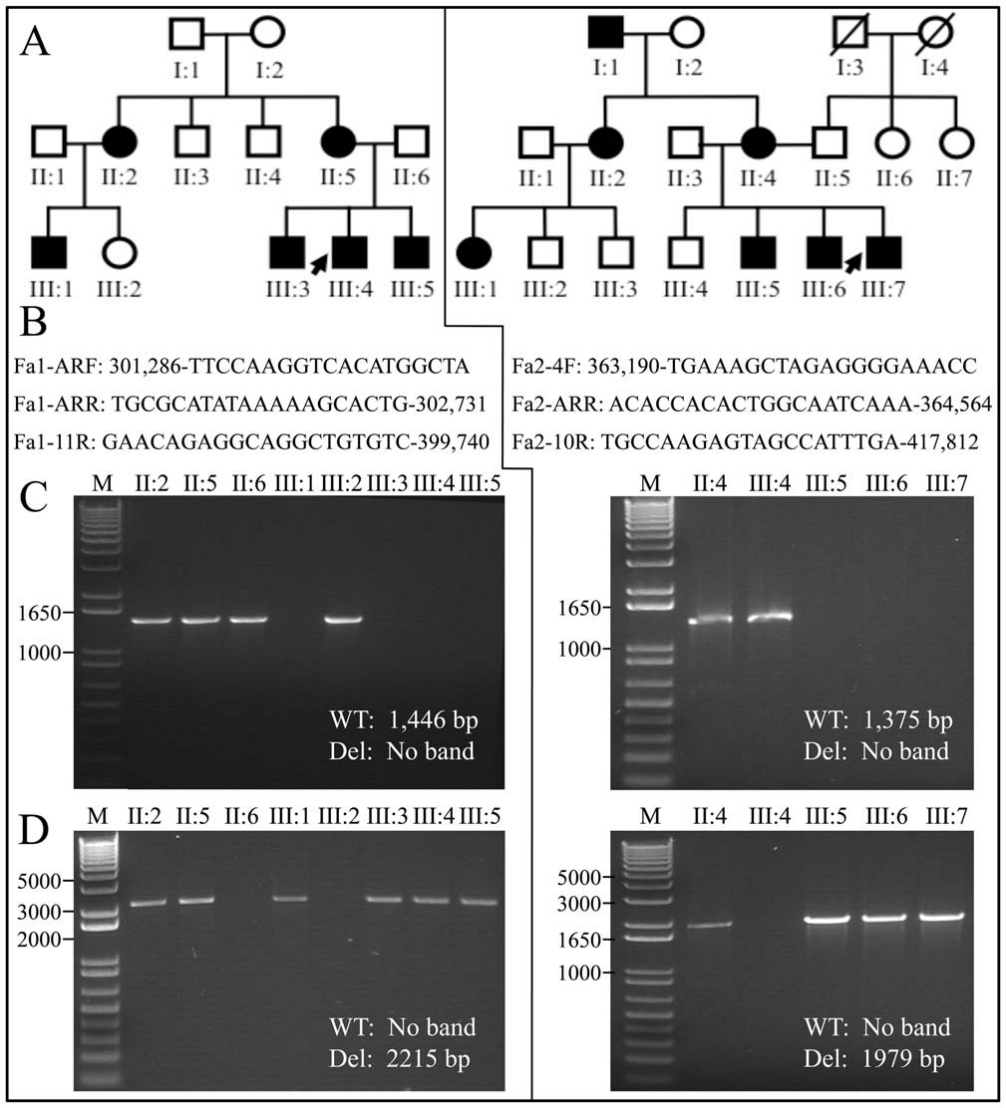
Correlating genotypes and affection status. ***A:*** Pedigrees for family 1 (left) and family 2 (right). ***B:*** PCR primers used to amplify across the deletion junctions. The numbers associated with the primers are the positions of the annealing sites in the *ARHGAP6* genomic reference sequence (NG_012494.1). The same forward primer was paired first with a reverse primer within the deleted region to specifically detect wild-type, and then with a primer that annealed on the other side of the deletion to specifically detect the defective allele. (The wild-type wouldn't amplify because the product is too large.). ***C:*** PCR amplification for wild-type X-chromosome. ***D:*** PCR amplification for X-chromosome with deletion. Normal males (XY) and females (XX) only show a product in the first amplification. Affected females (X*X) show a product in both amplifications. Affected males (X*Y) show only a product in the second amplification.

Mutation analyses using genomic DNA from the probands of families 1 and 2 were initiated, but none of the seven *AMELX* exons amplified with the appropriate primers. PCR primer pairs were designed to survey surrounding DNA sequence within *ARHGAP6*, the results of which narrowly defined the deleted regions in both families (Figs. S3, S4, S5, S6 and S7). Ultimately, we were able to amplify across the deletions in both families and determine the DNA sequences at the break points (Fig. 7). The deletions correlated with the enamel malformations in both families. All affected females were heterozygous for the *AMELX* deletion (X*X). The 96,240 bp deletion in *ARHGAP6* in family 1 (g.302534_398773del96240) was confined to intron 1 of that gene, but deleted all of *AMELX*. The 52,654 bp deletion in *ARHGAP6* in family 2 (g.363924_416577del52654insA) deleted the 3′ end of intron 1 including all of *AMELX*, exon 2, and a small part of the 5′ end of intron 2.

**Figure 8:**
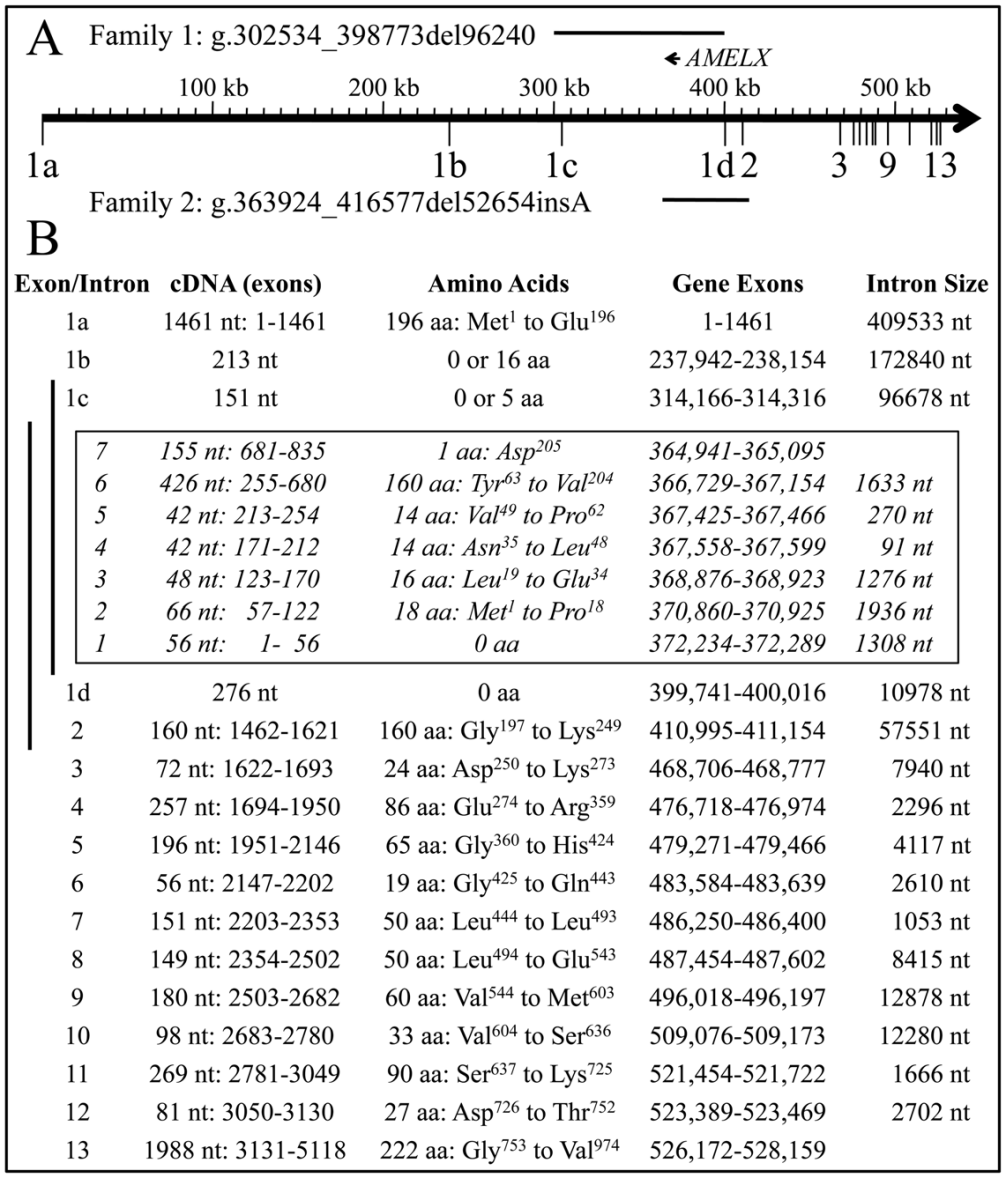
Summary of *ARHGAP6* gene structure and locations of deletions. ***A:*** Positions of the two *ARHGAP6* deletions. ***B:*** *ARHGAP6* gene structure inclusive of the four predicted promoters and *AMELX*.

To better assess the possible effects of these deletions on *ARHGAP6* expression we analyzed the DNA sequences 5′ to exon 2 on human and mouse *ARHGAP6* expressed sequence tags (ESTs). This analysis indicated that *ARHGAP6* expression is directed by at least 4 different promoters, each associated with a different exon 1 (exons 1a, 1b, 1c, and 1d). We identified one human EST and 4 mouse *ARHGAP6* ESTs that contained exon 1a; 5 human and 44 mouse ESTs with exon 1b; one human and 9 mouse ESTs with exon 1c; and one human and no mouse ESTs with exon 1d (Fig. S8). The structure of the *ARHGAP6* gene showing the positions of the alternative promoters, *AMELX*, and the deletions in families 1 and 2 is shown in Fig. 8.

**Figure 9:**
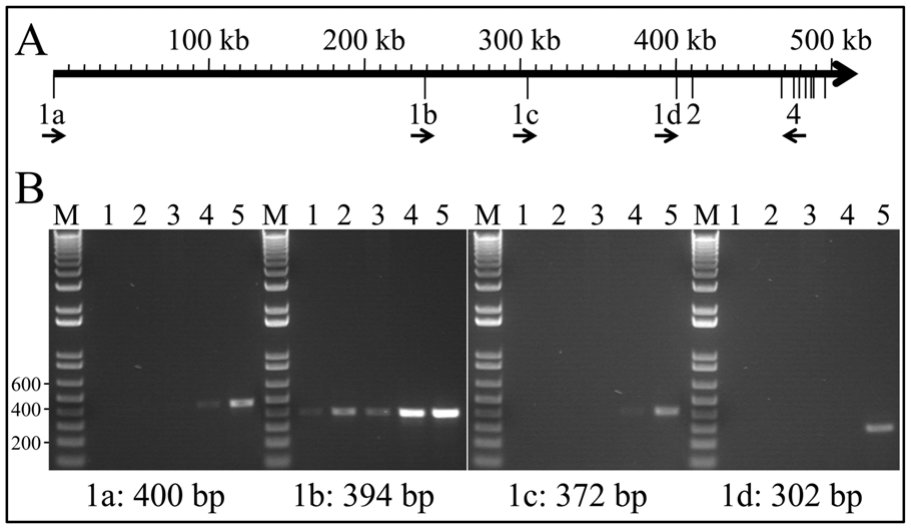
Alternative *ARHGAP6* promoter usage in secretory and maturation ameloblasts. ***A:*** Map of the 5′ end of *ARHGAP6* inclusive of the four promoters (indicated by exons 1a through 1d). Arrows indicate primer annealing sites for RT-PCR. ***B:*** PCR amplifcation products Key to RNA sources: Lanes 1: laser captured day 5 ameloblasts; Lanes 2: laser captured day 12 ameloblasts; Lanes 3: day 5 enamel organ epithelia; Lanes 4: day 12 enamel organ epithelia; Lanes 5: Positive PCR controls, spleen for 1a, lung for 1b, 1c, and 1d). These results indicate that *Arhgap6* is expressed almost exclusively from promoter 1b in developing molars, and mostly during the maturation stage.

To identify which *Arhgap6* promoters are used during enamel formation, we isolated mRNA from ameloblasts (by laser capture) and enamel organ epithelia (EOE) of mouse first molars at days 5 (secretory stage) and 12 (maturation stage), amplified them with primers specific for exons 1a through 1d, and analyzed the products on agarose gels stained with ethidium bromide (Fig. 9). No *Arhgap6* expression from promoters 1a and 1c was detected in secretory stage ameloblasts or EOE or maturation stage ameloblasts. Only trace expression was detected from these promoters in maturation stage EOE. *Arhgap6* expression from promoter 1b was low, but detectable, in secretory stage ameloblasts and EOE, and was noticably stronger in maturation stage ameloblasts and EOE. These results indicate that in developing mouse molars *Arhgap6* is expressed from promoter 1b, and mostly during the maturation stage.

*Arhgap6* exons 1a and 1b both contain predicted translation initiation sites in frame with the downstream coding sequence. Neither of these promoters were deleted in our families. Exon 2 however, was deleted in family 2. The skipping of exon 2 (160 nucleotides), which is entirely coding, would shift the reading frame and likely cause degradation of mRNA expressed from the truncated *Arhgap6* gene in family 2. Thus while both of our families have an X-chromosome lacking the amelogenin gene, the shortened X-chromosome in family 1 likely has a functional *ARHGAP6* gene, while the one in family 2 does not.

## Discussion

Amelogenin is a tooth-specific protein that accounts for almost 90% of the protein in secretory stage enamel [35]. The human expressed sequence tag database (Hs.654436), which does not include developing teeth, shows zero amelogenin transcripts out of 3,328,058 for normal tissues. The amelogenin gene is pseudogenized in vertebrates that have lost the ability to make teeth or enamel during evolution [36], [37], and tooth defects are the phenotype observed in families with *AMELX* mutations. Multiple amelogenins are expressed due to alternative splicing [14], [38], [39]. Following their secretion, amelogenins are processed by matrix metalloproteinase 20 (MMP20) into assorted cleavage products that accumulate throughout the matrix [40], [41]. Removal of amelogenin cleavage products from the matrix occurs primarily during the maturation stage [42], and requires the activity of kallikrein 4 [43], [44]. Human *AMELX* mutations produce a diversity of enamel phenotypes that seem to correlate with the position of the mutation [45]; however, a Tyr to His substitution in the N-terminal domain coding region of mouse *Amelx* caused extensive cytotoxicity because the ameloblasts failed to secrete the mutant protein, engorging the endoplasmic reticulum/Golgi apparatus. [46]. The AI phenotype in some cases must relate to secondary effects like cell pathology, rather than how enamel forms due to amelogenin insufficiency. Complete deletion of *AMELX* avoids this ambiguity and gives a clearer picture of how enamel forms when only trace amounts of amelogenin (from *AMELY*) are secreted. A remarkable observation in both of our families with *AMELX* deletions is that a thicker layer of enamel forms on the cusp tips and marginal ridges relative to the lateral tooth surfaces. The presence of this enamel cannot readily be explained by expression from *AMELY* as mice lack an amelogenin gene on the Y-chromosome, and yet *Amelx* null mice produce a thin enamel layer [47]. In contrast, *Enam* and *Ambn* null mice fail to make an enamel layer [48], [49]. These observations force a reconsideration of the possible mechanisms of dental enamel formation and favor a model in which enamelin and ameloblastin are essential components of a mineralization front apparatus along the secretory surfaces of the ameloblast cell membrane [50], whereas amelogenin serves other roles.

*AMELX* is nested within the large (>400 kb) first intron of *ARHGAP6* [51]. ARHGAP6 is expressed in many tissues, but at low levels. There are 28 *ARHGAP6* ESTs (Hs.435291) out of a total of 3,328,058 human ESTs for normal tissues. ARHGAP6 activates the GTPase activity of RhoA [27], which inhibits RhoA, a small G protein that regulates actin polymerization. RhoA activity appears to be essential for secretory stage enamel formation in that transgenic expression of enhanced green fluorescent protein (EGFP) fused to a dominant negative variant of RhoA (EGFP-RhoADN) in secretory stage ameloblasts resulted in the production of hypoplastic, pitted enamel [52]. ARHGAP6 may act in ways not mediated by RhoA by directly interacting with actin [27] or phospholipase C delta 1 (PLC-δ1) [53].

Both of our AI families had complete *AMELX* deletions, but the missing parts of *ARHGAP6* varied. The deletion in Family 1 removed *ARHGAP6* alternative promoters 1c and 1d. Our RT-PCR analyses showed that these promoters are not active in ameloblasts, so it is unlikely that this deletion affects *ARHGAP6* expression in developing teeth. The deletion in Family 2 removed exon 2 of *ARHGAP6*, which should have precluded expression of the ARHGAP6 protein. It seems likely then that Family 1 had an *AMELX* defect only, while Family 2 had a combined *AMELX* and *ARHGAP6* defect. The enamel phenotypes in both families were similar with a characteristic snow-capped appearance caused by the deposition of a relatively thick layer of enamel on the cusp tips, the buccal-occlusal and lingual-occlusal cusp slopes, and marginal ridges. The enamel defects were severe, but there was not a complete absence of enamel. The proband of Family 2 seemed to have less contrasting enamel on radiographs and greater surface roughness than the affected members of Family 1. The enamel phenotypes in these two families are remarkably similar given the broader phenotypic variation observed among the many persons with defined *AMELX* mutations (Fig. S1). We suspect that the minor variations in enamel phenotype between our two families reflects the natural range of phenotypic variation in persons lacking *AMELX*, but the suspicion that an absence of ARHGAP6 is the cause of the increased surface roughness cannot be excluded given the minor differences in enamel phenotype observed in *Amelx* null and *Amelx*/*Arhgap6* double null mice [26]. Despite the possibility that defects in *ARHGAP6* could modify the *AMELX* null phenotype, *ARHGAP6* defects alone apparently do not cause enamel malformations. *Arhgap6* null mice showed no enamel defects [26]. *AMELX* mutations are consistently found in human kindreds with X-linked AI, suggesting there is no second X-linked gene that causes AI, as this would lead to the accumulation of unsolved X-linked AI cases without *AMELX* mutations, which is not observed.

## Supporting Information

Figure S1 ***AMELX*** **disease-causing mutations.**
(DOC)

Figure S2 **Tooth H from the proband of family 2 before it was processed for SEM analyses.**
(DOC)

Figure S3 **PCR amplification of the 7** ***AMELX*** **exons.**
(DOC)

Figure S4 **PCR amplifications in intron 1 and exons 2 through 7 of** ***ARHGAP6*** **.**
(DOC)

Figure S5 **PCR amplifications in family 1.**
(DOC)

Figure S6 **PCR amplifications in family 2.**
(DOC)

Figure S7 **More PCR amplifications in family 2.**
(DOC)

Figure S8 **Mouse (A) and Human (B) ARHGAP6 expressed sequence tags.**
(DOC)
