# Supplementary material for: Amelogenesis Imperfecta in Two Families with Defined AMELX Deletions in ARHGAP6
Source: PLoS One. 2012 Dec 14;7(12):e52052. doi: 10.1371/journal.pone.0052052 (PMC3522662; doi:10.1371/journal.pone.0052052)
Supplement: Figure S1 — AMELX disease-causing mutations. (DOC) [file pone.0052052.s001.doc]

**
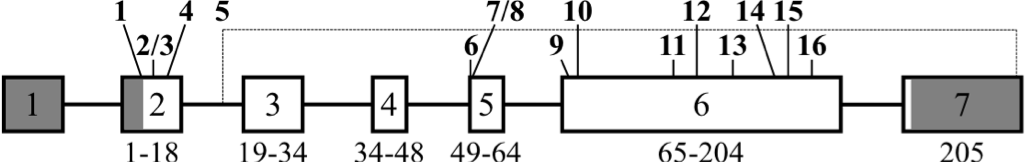
**

**# Gene cDNA Protein References**

1 g.1378T>C c.2T>C p.M1T

2 g.1387G>C c.11G>C p.W4S

3 g.1387G>A c.11G>A p.W4X

4 g.1390_1398del9 c.14_22del9 p.I5-A8delinsT

5 g.2525_7247del4723 c.55-842_665del4723 no protein

6 g.4831C>T c.152C>T p.T51I

7 g.(4832_4834)delC c.(152_155)delC p.P52LfsX53

8 g.4834G>C c.155C>G p.P52R

9 g.4090delC c507delC p.P173LfsX16

9 g.5157C>A c.208C>A p.P70T

10 g.5179 c.230A>T p.H77L

11 g.(5333_5335)delC c.(384_385)delC p.H129fsX187

12 g.(5367_5369)delC c.(418_420)delC p.Y141fsX187

13 g.(5421_5422)delC c.(472_473)delC p.P158HfsX187

14 g.(5466_5477)delC c.(517_518)delC p.P173LfsX16

15 g.(5487_5490)delC c.(538_541)delC p.L181CfsX187

16 g.5520G>T c.571G>T p.E191X.


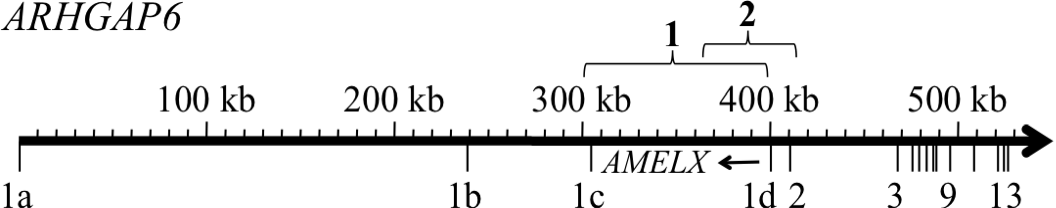


Family 1: g.302534_398773del96240 no amelogenin ARHGAP6

Family 2: g.363924_416577del52654insA no amelogenin No ARHGAP6

**Figure S1.** *AMELX* disease-causing mutations. ***Top:*** *AMELX* gene structure: numbered boxes are exons with the range of amino acids encoded by it. Mutation 5 is the deletion indicated by dashed lines. The gene numbers start from the first nucleotide of the *AMELX* reference sequence NG_012040.1. The cDNA numbers start from the translation initiation site of *AMELX* cDNA reference sequence NM_182680.1. **Bottom:** Deletions of *AMELX* in *ARHGAP6*. Deletion 1 includes *ARHGAP6* promoter 1c and *AMELX*. Deletion 2 includes *ARHGAP6* promoter 1d, *AMELX* and *ARHGAP6* exon 2. Deletion of exon 2 eliminates expression of *ARHGAP6*. The references of the reported *AMELX* mutations are listed.

**References:**

1. Kim J-W, Simmer JP, Hu YY, Lin BP-L, Boyd C, et al. (2004) Amelogenin p.M1T and p.W4S mutations underlying hypoplastic X-linked amelogenesis imperfecta. J Dent Res 83: 378-383.

2. Sekiguchi H, Kiyoshi M, Yakushiji M (2001) DNA diagnosis of X-linked amelogenesis imperfecta using PCR detection method of the human amelogenin gene. Dent Japan 37: 109-112.

3. Lagerstrom-Fermer M, Nilsson M, Backman B, Salido E, Shapiro L, et al. (1995) Amelogenin signal peptide mutation: correlation between mutations in the amelogenin gene (AMGX) and manifestations of X-linked amelogenesis imperfecta. Genomics 26: 159-162.

4. Lagerström M, Dahl N, Iselius L, Bäckman B, Pettersson U (1990) Mapping of the gene for X-linked *amelogenesis imperfecta* by linkage analysis. Am J Hum Genet 46: 120-125.

5. Lagerström M, Dahl N, Nakahori Y, Nakagome Y, Backman B, et al. (1991) A deletion in the amelogenin gene (AMG) causes X-linked amelogenesis imperfecta (AIH1). Genomics 10: 971-975.

6. Lench NJ, Winter GB (1995) Characterisation of molecular defects in X-linked amelogenesis imperfecta (AIH1). Hum Mutat 5: 251-259.

7. Aldred MJ, Crawford PJ, Roberts E, Thomas NS (1992) Identification of a nonsense mutation in the amelogenin gene (AMELX) in a family with X-linked amelogenesis imperfecta (AIH1). Hum Genet 90: 413-416.

8. Lench NJ, Brook AH, Winter GB (1994) SSCP detection of a nonsense mutation in exon 5 of the amelogenin gene (AMGX) causing X-linked amelogenesis imperfecta (AIH1). Hum Mol Genet 3: 827-828.

9. Kida M, Sakiyama Y, Matsuda A, Takabayashi S, Ochi H, et al. (2007) A novel missense mutation (p.P52R) in amelogenin gene causing X-linked amelogenesis imperfecta. J Dent Res 86: 69-72.

10. Wright JT, Torain M, Long K, Seow K, Crawford P, et al. (2011) Amelogenesis Imperfecta: Genotype-Phenotype Studies in 71 Families. Cells Tissues Organs 194: 279-283.

11. Lee K-E, Lee S-K, Jung S-E, Song SJ, H. CS, et al. (2011) A novel mutation in the AMELX gene and mjltiple corown resorptions. Eur J Oral Sci 119 (Suppl.1): 324-328.

12. Collier PM, Sauk JJ, Rosenbloom SJ, Yuan ZA, Gibson CW (1997) An amelogenin gene defect associated with human X-linked amelogenesis imperfecta. Arch Oral Biol 42: 235-242.

13. Hart S, Hart T, Gibson C, Wright JT (2000) Mutational analysis of X-linked amelogenesis imperfecta in multiple families. Arch Oral Biol 45: 79-86.

14. Ravassipour DB, Hart PS, Hart TC, Ritter AV, Yamauchi M, et al. (2000) Unique enamel phenotype associated with amelogenin gene (AMELX) codon 41 point mutation. J Dent Res 79: 1476-1481.

15. Chan HC, Estrella NM, Milkovich RN, Kim JW, Simmer JP, et al. (2011) Target gene analyses of 39 amelogenesis imperfecta kindreds. Eur J Oral Sci 119: 311-323.

16. Hart PS, Aldred MJ, Crawford PJ, Wright NJ, Hart TC, et al. (2002) Amelogenesis imperfecta phenotype-genotype correlations with two amelogenin gene mutations. Arch Oral Biol 47: 261-265.

17. Sekiguchi H, Alaluusua S, Minaguchi K, Yakushiji M (2001) A new mutation in the amelogenin gene causes X-linked amelogenesis imperfecta. J Dent Res 80: 617.

18. Greene SR, Yuan ZA, Wright JT, Amjad H, Abrams WR, et al. (2002) A new frameshift mutation encoding a truncated amelogenin leads to X-linked amelogenesis imperfecta. Arch Oral Biol 47: 211-217.

19. Kindelan SA, Brook AH, Gangemi L, Lench N, Wong FS, et al. (2000) Detection of a novel mutation in X-linked amelogenesis imperfecta. J Dent Res 79: 1978-1982.
