# Supplementary figures and images for: Amelogenesis Imperfecta in Two Families with Defined AMELX Deletions in ARHGAP6
Source: PLoS One. 2012 Dec 14;7(12):e52052. doi: 10.1371/journal.pone.0052052 (PMC3522662; doi:10.1371/journal.pone.0052052)

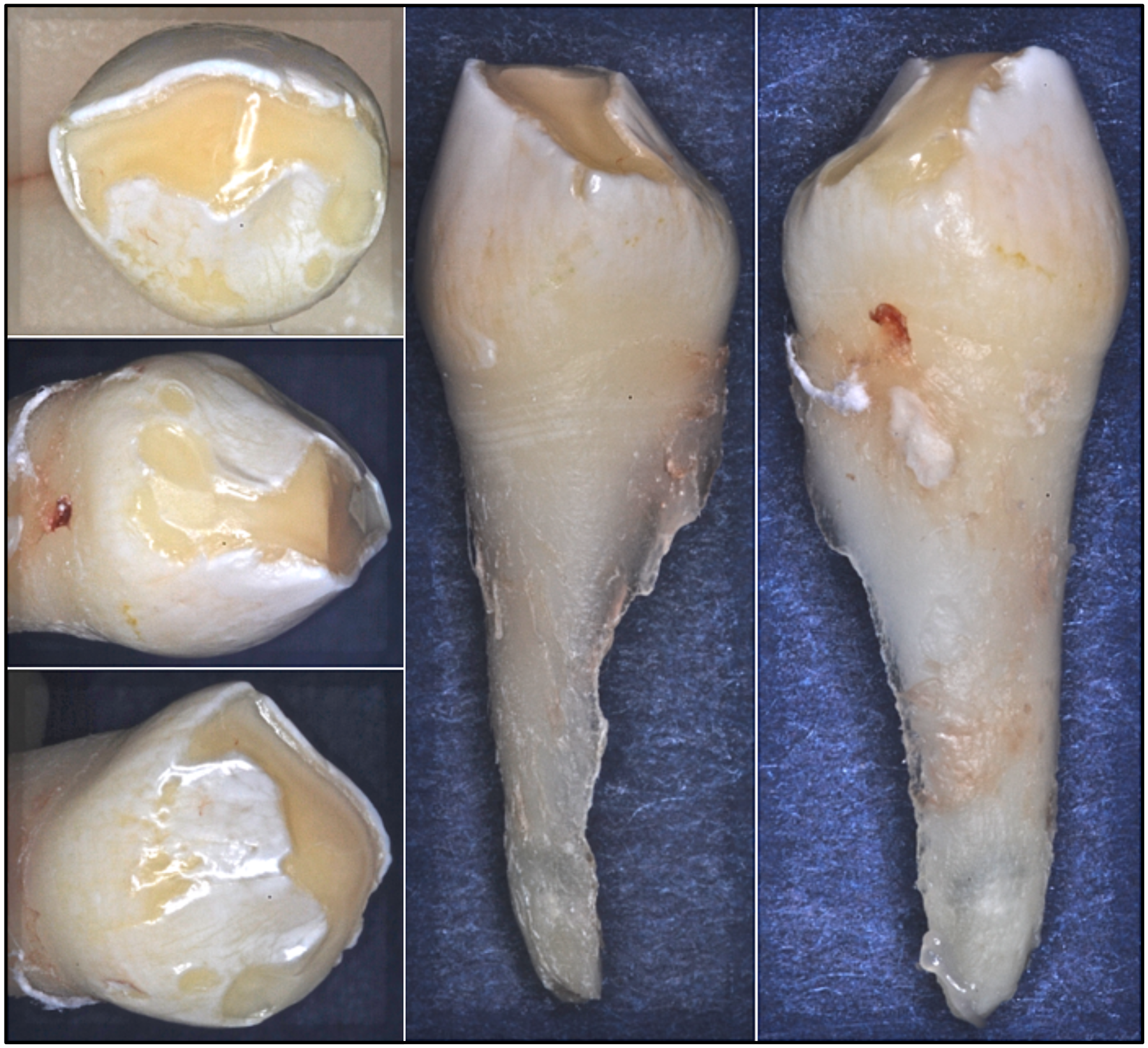


**Figure S2.** Tooth H from the proband of family 2 before it was processed for SEM analyses.

Supplement: Figure S2 — Tooth H from the proband of family 2 before it was processed for SEM analyses. (DOC) [file pone.0052052.s002.doc]
