# Supplementary material for: Amelogenesis Imperfecta in Two Families with Defined AMELX Deletions in ARHGAP6
Source: PLoS One. 2012 Dec 14;7(12):e52052. doi: 10.1371/journal.pone.0052052 (PMC3522662; doi:10.1371/journal.pone.0052052)
Supplement: Figure S3 — PCR amplification of the 7 AMELX exons. (DOC) [file pone.0052052.s003.doc]

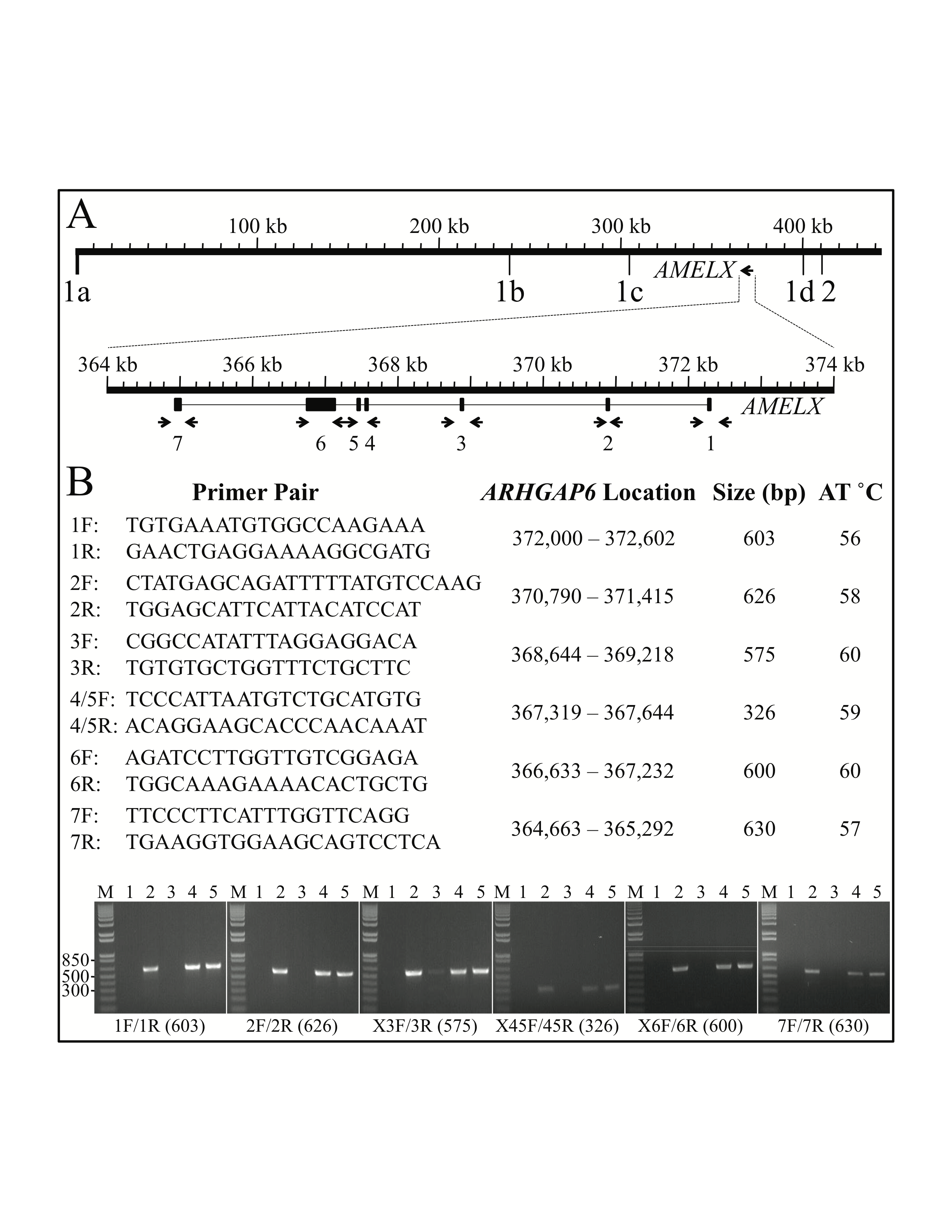


**Figure S3.** PCR amplification of the 7 *AMELX* exons. ***A:*** Genetic map of the first intron of *ARHGAP6*, which contains *AMELX* (leftward point arrow at ~370 kb), showing the positions of four alternative first exons (1a through 1d) and exon 2 (at ~410 kb). Expansion of the region between 364 kb and 374 kb of *ARHGAP6* shows the nested *AMELX* exons and the annealing sites of primers used to amplify the exons for mutation analyses. ***B:*** The primer pairs used for mutational analyses, the sizes of their amplification products, the locations of the amplification products in the *ARHGAP6* genomic reference sequence (NG_012494.1), and the annealing temperatures used in the PCR reactions. The amplification reactions included a 5 min denaturation at 94 ˚C followed by 35 cycles at 94 ˚C for 30 s, annealing for 1 min, extension at 72 ˚C for 90 s, followed by a final extension for 7 min. ***C:*** Ethidium bromide-stained agarose gels showing the amplification products for family 2. Lanes 1: III:7, Lanes 2: II:4, Lanes 3: III:6, Lane 4: normal male, Lane 5: normal female. These analyses demonstrated that *AMELX* was completely deleted in the probands from both families.
