# Supplementary material for: Amelogenesis Imperfecta in Two Families with Defined AMELX Deletions in ARHGAP6
Source: PLoS One. 2012 Dec 14;7(12):e52052. doi: 10.1371/journal.pone.0052052 (PMC3522662; doi:10.1371/journal.pone.0052052)
Supplement: Figure S4 — PCR amplifications in intron 1 and exons 2 through 7 of ARHGAP6 . (DOC) [file pone.0052052.s004.doc]

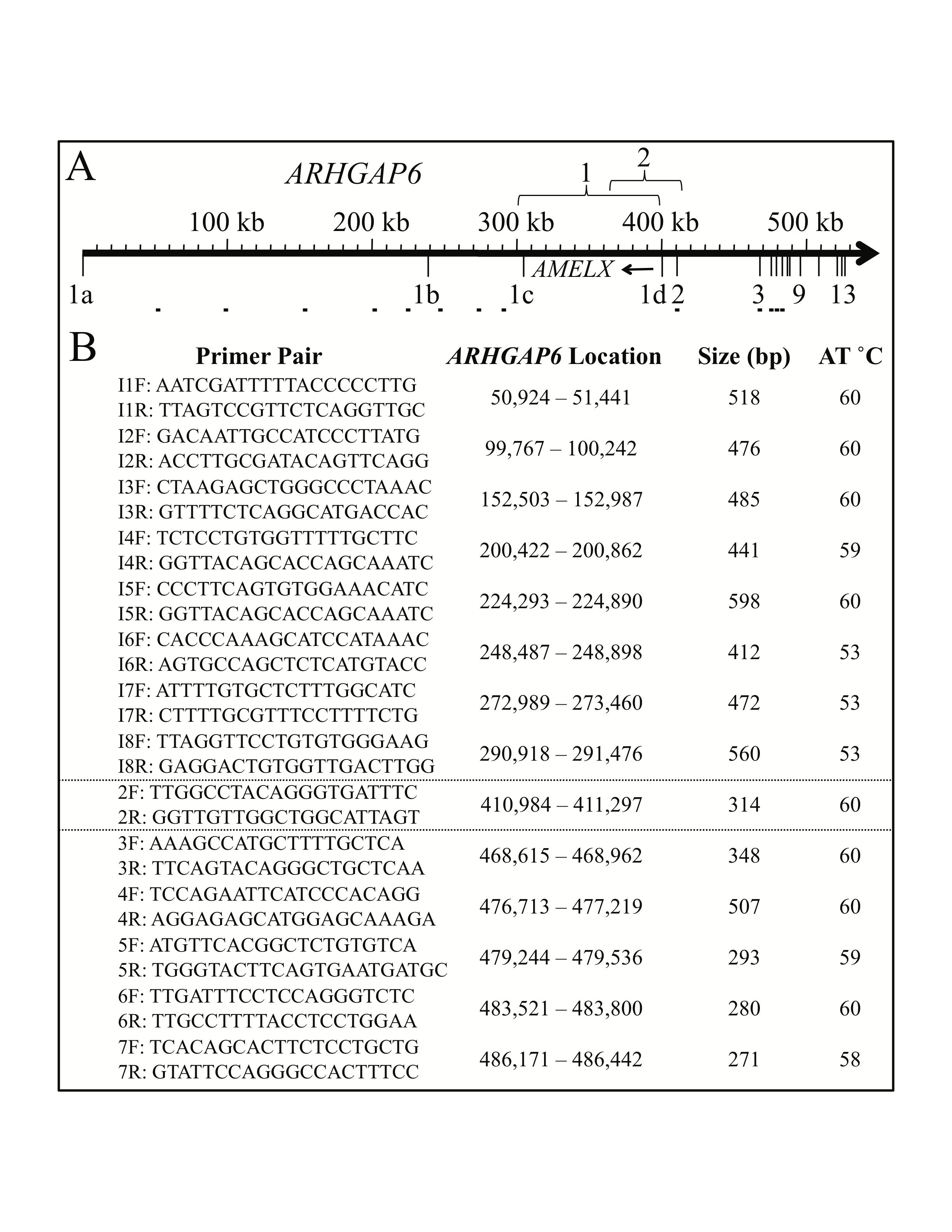


**Figure S4.** PCR amplifications in intron 1 and exons 2 through 7 of *ARHGAP6*. ***A:*** Gene map showing the positions of the four alternative first exons (1a, 1b, 1c, and 1d) and exons 2 through 13. The numbered brackets indicate the deletions in family 1 and family 2. Small bars below the map are the sites amplified to roughly define the extents of the deletions. ***B:*** The primer pairs used for the PCR analyses, the sizes of their amplification products, locations of the amplification products in the *ARHGAP6* genomic reference sequence (NG_012494.1), and the annealing temperatures used the in PCR reactions. Primer numbers indicate the exon that the primers anneal to; an “I” before the primer designation indicates the annealing site is in intron 1. The dashed lines delineate the only primer pair that did not give an amplification product: the exon 2 set, which did not amplify genomic DNA from the proband of family 2. These analyses narrowed down the positions of the deletions.
