# Supplementary material for: Amelogenesis Imperfecta in Two Families with Defined AMELX Deletions in ARHGAP6
Source: PLoS One. 2012 Dec 14;7(12):e52052. doi: 10.1371/journal.pone.0052052 (PMC3522662; doi:10.1371/journal.pone.0052052)
Supplement: Figure S5 — PCR amplifications in family 1. (DOC) [file pone.0052052.s005.doc]

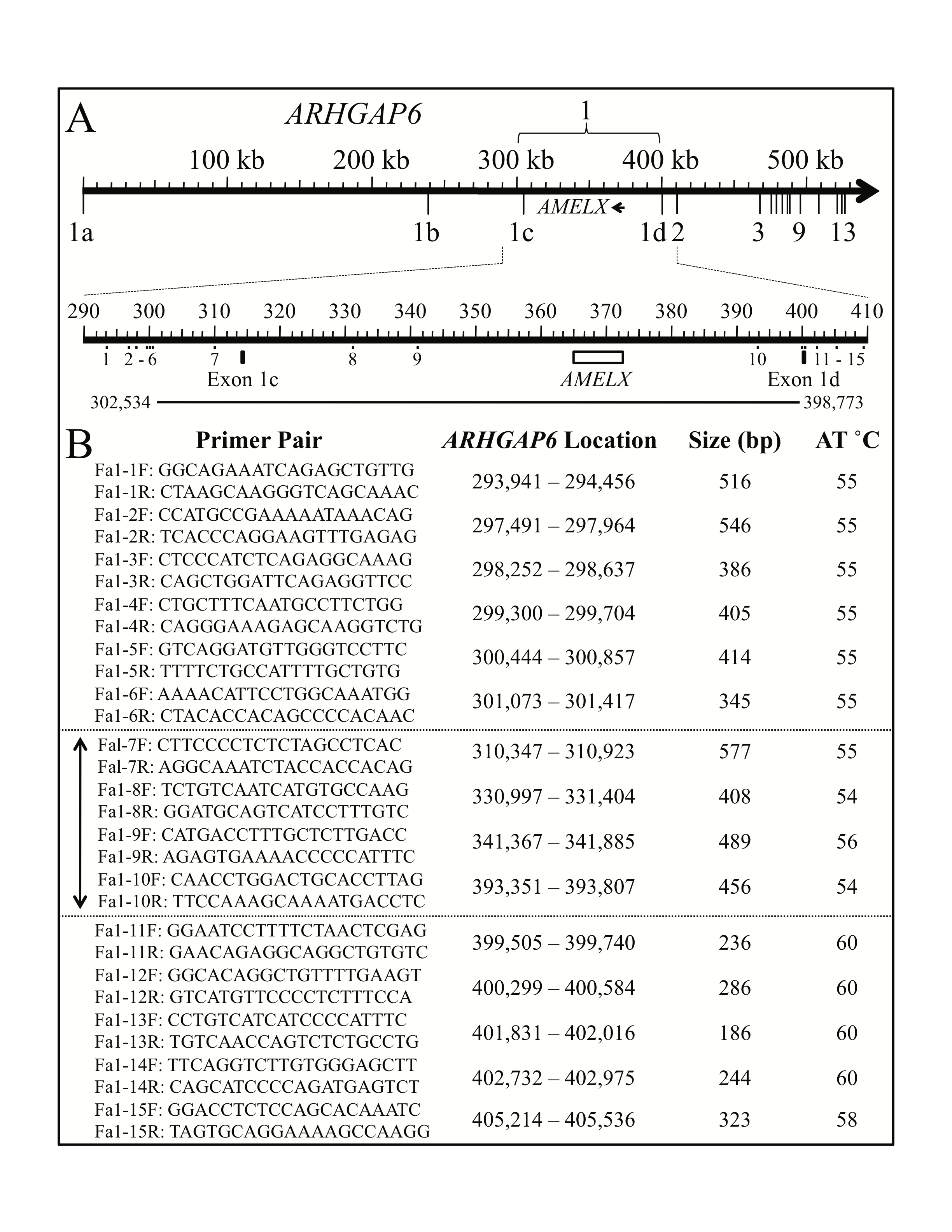


**Figure S5.** PCR amplifications in family 1. ***A:*** *ARHGAP6* gene map showing the position of the deletion in family 1. The region between 290 and 410 kbp is expanded to show the 15 segments, indicated by small bars, that were amplified to more precisely define the borders of the deletion. ***B:*** The primer pairs used for the PCR analyses, the sizes of their amplification products, the locations of the amplification products in the *ARHGAP6* genomic reference sequence (NG_012494.1), and the annealing temperatures used in the PCR reactions. The dashed lines delineate the primer pairs that did not give an amplification product. Note that only *ARHGAP6* exon 1c lies within the deleted segment.
