# Supplementary material for: Amelogenesis Imperfecta in Two Families with Defined AMELX Deletions in ARHGAP6
Source: PLoS One. 2012 Dec 14;7(12):e52052. doi: 10.1371/journal.pone.0052052 (PMC3522662; doi:10.1371/journal.pone.0052052)
Supplement: Figure S6 — PCR amplifications in family 2. (DOC) [file pone.0052052.s006.doc]

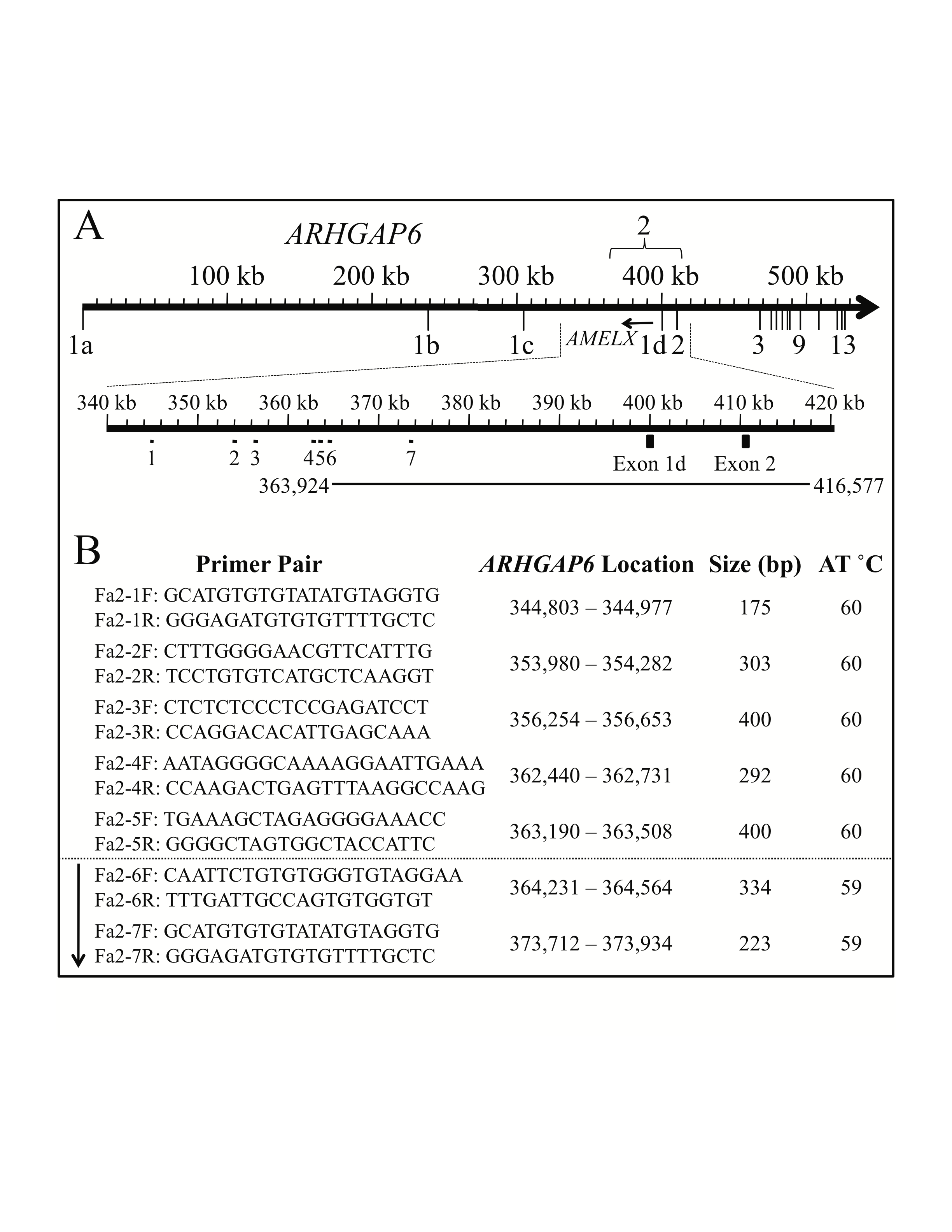


**Figure S6.** PCR amplifications in family 2. ***A:*** *ARHGAP6* gene map showing the position of the deletion in family 2. The region between 340 and 420 kbp is expanded to show the 7 segments that were amplified to more precisely define the 5’ border of the deletion in family 2. ***B:*** The primer pairs used for the PCR analyses, the sizes of their amplification products, locations of the amplification products in the *ARHGAP6* genomic reference sequence (NG_012494.1), and the annealing temperatures used in the PCR reactions. The dashed line delineates the two primer pairs that did not give an amplification product.
