# Supplementary material for: Amelogenesis Imperfecta in Two Families with Defined AMELX Deletions in ARHGAP6
Source: PLoS One. 2012 Dec 14;7(12):e52052. doi: 10.1371/journal.pone.0052052 (PMC3522662; doi:10.1371/journal.pone.0052052)
Supplement: Figure S8 — Mouse (A) and Human (B) ARHGAP6 expressed sequence tags. (DOC) [file pone.0052052.s008.doc]

**A**

**Mouse ESTs extending to Exon 1, 1a, 1b, or 1c**

**Contain Exon 1a**

adult male corpus striatum: BB595293.1

activated spleen: BY557428.1; BY567429.1

adult inner ear: BY589053.1

**Contain Exon 1b**

B6-derived CD11 +ve dendritic cells: BB850442.1; BY195373.1; BY200227.1; BY201317.1; BY201593.1; BY203405.1; BY203730.1; BY203943.1; BY203998.1; BY209591.1; BY543948.1; BY544668.1; BY749611.1; BY749761.1; BY749761.1; BY750027.1

NOD-derived CD11c +ve dendritic cells: BY183843.1; BY748487.1; BY179323.1; BY180258.1; BY175742.1; BY191097.1

13 days embryo male testis: BB621563.1

16 days embryo head: BB648246.1; BB648776.1; BB648728.1; BB648130.1

17 days embryo heart: BY062053.1

17.5 days embryo whole body: BY772197.1; BY789656.1; BY140596.1; BY785956.1; BY140361.1

adult male spinal cord: BB634009.1

bone marrow mast cells: BY228202.1; BY228509.1

lung RCB-0558 LLC cDNA: BY012179.1; BY012109.1; BY009409.1; BY010541.1

adult inner ear: BB850444.1; BB850165.1; BY236133.1; BY229891.1

**Contain Exon 1c**

lung RCB-0558 LLC**:** BY734296.1; BY013989.1; BY013329.1; BY014201.1; BY011501.1; BY012571.1; BB818490.1; BB817061.1

pooled cell lines**:** BB859914.1

**Contain Exon 1d:** None

**B**

**Human ESTs extending to Exon 1, 1a, 1b, or 1c**

**Contain Exon 1a**

DB281876.1 (uterus)

**Contain Exon 1b**

HY103225.1 (thymus); HY018118.1 (testis); DR006187.1 (prostate);

BQ930645.1 (sciatic nerve); BM543771.1 (unknown)

**Contain Exon 1c**

DC409127.1 (thymus)

**Contain Exon 1d**

DA542981.1 (coronary artery smooth muscle cells)

**Figure S8.** Mouse (A) and Human (B) ARHGAP6 expressed sequence tags that show the presence of four alternative first exons that indicate the use of four different promoters that are conserved between these two species.
